# Development and psychometric testing of the Family Functioning Questionnaire in Rehabilitation (FFQR)

**DOI:** 10.3906/sag-1909-93

**Published:** 2019-12-16

**Authors:** Hatice ABAOĞLU, Esra AKI

**Affiliations:** 1 Department of Occupational Therapy, Faculty of Health Sciences, Hacettepe University, Ankara Turkey

**Keywords:** Family, rehabilitation, children with disabilities, questionnaire design, reliability and validity

## Abstract

**Background/aim:**

The present study aimed to develop a reliable and valid assessment tool for measuring family functioning in rehabilitation.

**Materials and methods:**

Semistructured interviews were performed with 100 rehabilitation professionals working in pediatrics to identify the feature to be measured. The items determined with the qualitative analysis of the data were presented to 14 experts and content validity was provided. The questionnaire created based on the judgments of the experts was administered to 440 parents of children with special needs.

**Results:**

After validity and reliability analysis, the final version of the questionnaire comprised 48 items with four factors identified as awareness, attitude and behavior, social participation, and engagement in rehabilitation. These factors explained 49.94% of the total variance and the factor loadings ranged from 0.492 to 0.773. Internal consistency reliability calculated with the Cronbach alpha coefficient was found to be 0.943. The test-retest reliability coefficient between the two administrations with a two-week interval was found as 0.772.

**Conclusion:**

The findings of the study showed that the newly developed Family Functioning Questionnaire in Rehabilitation met the criteria for examining the role of families of children with special needs in rehabilitation programs and had adequate psychometric properties.

## 1. Introduction

Children with special needs are children who are at risk or have physical, developmental, behavioral, or emotional conditions that require health and related services in a type or amount that is beyond the needs of children in general [1]. To meet the needs of care, education, and rehabilitation of these children who depend on their families in every aspect of daily life is a long, tedious, and challenging process [2,3]. Having a child with special needs affects the roles and responsibilities of the family. Family members try to adapt to the stress, physical effort, role and identity changes, and financial and psychological problems that arise as a result of the child’s health status. They also undertake responsibilities such as interacting with various professionals including physicians, physiotherapists, occupational therapists, and special education specialists; providing environmental modifications, equipment, or assistive devices; and supporting skill training and other intervention programs [3–5].

Families know their children well and want the best for them. A supportive family and society positively influence the child’s functioning, quality of life, and social participation. Parents are constantly in contact with health professionals and are part of the team in choosing, implementing, and maintaining education and treatment programs for children with special needs. Especially in recent years, it has been emphasized that family has an important role in understanding and meeting the needs and abilities of children with special needs [5,6]. 

Family-centered approaches that increase the quality of life of the child, as well as the quality of life of the family, have gradually become the focus of pediatric rehabilitation. Family-centered practice is a dynamic process that adapts to the changing situation, needs, and priorities of the child and the family. These interventions enhance the child’s physical, emotional, social, and cognitive functions; promote meaningful activities and social participation and improve engagement in treatment programs; and recognize the child as a family member and acknowledge the influence of other family members [7–9].

In the International Classification of Functioning, Disability, and Health (ICF), developed by the World Health Organization for providing comprehensive information on health and health-related issues, it is emphasized that assessments for rehabilitation interventions should have a holistic view that takes into account the relationship of the individual and the individual’s sociocultural environment. According to the ICF, functioning is a part of the ongoing dynamic developmental process through interaction with the family [10]. To be able to identify the factors that can affect the individual and the rehabilitation process, the evaluation of the whole family is important [11]. Family-centered assessments aim to identify the strengths of the family and the child. Priorities, values, expectations, and the needs of the families are the focus of evaluation. In this process, family members have the opportunity to observe their attitudes towards their children [12].

Family support is important in ensuring the participation of children with special needs in school and community life. The family contributes to health and well-being by providing support and transportation, influencing the success of long-term rehabilitation, and enabling positive living in spite of the disability. For this reason, family-centered assessments should take into account the importance of the role of the family in rehabilitation [13,14]. Previous studies have presented some assessment tools for investigating physical, emotional, or psychological influences and the needs and social participation of parents after having a child with special needs [15–18]. In addition, there are commonly used generic measures assessing various aspects of family functioning such as the Impact on Family Scale [19], which measures parents’ perception of the effects of the child’s condition on family life, and the Family Environment Scale [20], which is used to assess the family environment from different perspectives within the family. However, to the best of our knowledge, there is a need for an objective, valid, and reliable tool that can measure the extent to which the family contributes to rehabilitation. Therefore, the objectives of the present study were to develop an assessment tool that measures family functioning in rehabilitation and to establish its psychometric properties. 

## 2. Materials and methods

### 2.1. Study design

In the present study, a questionnaire which is specific to Turkish society was developed for the families of children with special needs. The study protocol was performed following the ethical codes of the World Medical Association (Declaration of Helsinki) and was approved by the Hacettepe University Noninterventional Clinical Research Ethics Board (Approval no: GO 14/416, Date: 05.11.2014). All participants provided written informed consent. This study was designed with an exploratory sequential mixed methods approach integrating qualitative and quantitative research and data. The mixed methods research design is particularly useful when developing and testing an instrument if there is no available measure or the existing ones poorly represent the phenomenon [21]. 

### 2.2. Participants 

Three different sample groups were included in the present study. To determine the feature to be measured the first sample included 100 rehabilitation professionals working in the field of pediatrics, including physiotherapists, psychologists, child development specialists, special education teachers, occupational therapists, a social worker, and a physician. Secondly, to review and confirm the content validity of the draft questionnaire a team of experts consisting of fourteen academicians with expertise in health sciences was consulted. Finally, the third sample comprised 440 parents of children with special needs who applied to the Hacettepe University Faculty of Health Sciences Department of Physical Therapy and Rehabilitation and Occupational Therapy in Ankara, Turkey. Inclusion criteria for parents were to have a child with special needs between the ages of 1 and 18 years, to continue a rehabilitation program for at least one year, and to participate voluntarily after being informed verbally and in writing about the research. Exclusion criteria were not being able to establish communication and cooperation and not being a primary caregiver of a child with special needs. 

### 2.3. Procedure

#### 2.3.1. Phase 1: Item generation

A literature review and semistructured interviews were used to develop the items of the Family Functioning Questionnaire in Rehabilitation (FFQR). The literature was reviewed to identify the role of the family in rehabilitation and related domains. After the literature review, 5 initial domains were determined for the interviews: environment (home, school environment, rehabilitation centers, other family members living at home, relatives, acquaintances, friends, physical and social environment), time (the time spent for children, such as treatment sessions), communication (communication with health professionals, children with special needs, other people), support (socioeconomic level, educational activities, security, social participation), and other issues. The initial items of the FFQR were generated by conducting semistructured interviews with 100 rehabilitation professionals. Data obtained from the interviews were recorded and analyzed through thematic analysis. 

#### 2.3.2. Phase 2: Expert views and content validity

As a result of the qualitative analysis of the obtained data, an item pool with 121 statements was created. Fourteen experts with sufficient knowledge and experience in the rehabilitation of children with special needs evaluated the appropriateness of the draft scale. Expert assessments included views on whether the items represented the feature to be measured, whether it was expressed simply and clearly, and whether it would be understood by the target group. With the feedback received from the experts, the comprehensibility, usefulness, and suitability of the items were reviewed and necessary adjustments were made. 

In addition to the qualitative content validity method, the content validity ratio (CVR) was used to make the expert opinions digitized and statistically interpretable. For this purpose, experts were asked to rate each item using a three-point ordinal scale (1: not necessary, 2: useful but not essential, 3: essential). The CVR was developed by Lawshe [20] as a statistical value that reflects whether each item is included in the scale and its varies between 1 and –1. It is calculated with the following formula: CVR = (ne – N/2)/(N/2), in which ne is the number of experts indicating “essential” and n is the total number of experts. Once the CVR of each item is computed, the items with positive CVRs are compared with the minimum CVR, which is determined by the number of experts at a certain level of significance. Only the items meeting the minimum CVR value remain in the scale. In the present study, since the expert team was composed of 14 members, a minimum CVR of 0.51 was required at the 5% level of significance. After the retained items were identified, the content validity index (CVI), the mean of the CVR values of those items, was computed for the whole test [22].

#### 2.3.3. Phase 3: Administration of the questionnaire

The initial version of the FFQR, consisting of 88 items, was administrated to 440 parents of children with special needs. Participants responded to each question using a five-point Likert scale ranging from 1, ‘strongly disagree,’ to 5, ‘strongly agree.’ In addition to the FFQR, a demographics form was used to gather typical demographic information about the children and their families (e.g., age, sex, diagnosis of the child).

### 2.4. Data analyses

An item analysis was performed to assess whether the individual items contributed to the total questionnaire. Item-to-total correlation coefficients and the reliability coefficients if item deleted were computed for item analysis.

Exploratory factor analysis (EFA) was used to evaluate the structural validity. Bartlett’s test of sphericity and the Kaiser–Meyer–Olkin (KMO) test of sampling adequacy were calculated to determine the factorability of the data. After the data were found suitable for factor analysis, principal component analysis (PCA) with varimax rotation was used to examine the factor structure of the questionnaire. Factors with eigenvalues greater than 2 were considered significant.

Internal consistency and test-retest analyses were conducted for the reliability of the questionnaire. The Cronbach alpha coefficient was used to evaluate internal consistency. To confirm the test-retest reliability, the Pearson correlation coefficient was calculated between two administrations.

SPSS 23.0 for Windows was used for statistical analyses and the significance level was defined as P < 0.05.

## 3. Results

In this study, which aimed to develop a questionnaire that measures family functioning in rehabilitation, a total of 100 rehabilitation professionals were interviewed to determine the feature to be measured. Table 1 provides the demographic data of the participants who took part in interviews (age, sex, profession, academic qualification, and years of experience). 

**Table 1:** Demographic information about rehabilitation professionals.

Demographic variable	X ± SD
Age	29.41 ± 6.55
	n (%)
Sex Female Male	61 (61%) 39 (39%)
Profession Physiotherapist Child development specialist Psychologist Special education teacher Occupational therapist Social worker Physician	66 (66%) 20 (20%) 6 (6%) 4 (4%) 2 (2%) 1 (1%) 1 (1%)
Academic qualification Bachelor Master Doctorate	58 (58%) 34 (34%) 8 (8%)
Years of experience 1–5 6–10 11–15 16–20 ≥21	51 (51%) 23 (23%) 13 (13%) 4 (4%) 9 (9%)

After the expert views were obtained, the CVRs were calculated for each item and those with negative and zero values were first excluded from the scale. When the items with positive CVRs were compared with the minimum CVR (0.51) determined according to the number of experts, 33 items with CVRs below 0.51 were excluded from the scale. The remaining 88 items were renumbered and a draft form of the FFQR was created. The CVI of the scale was calculated as the average of the CVR values for the remaining items. The CVI was 0.75, indicating that the FFQR had content validity [23].

The 88-item draft scale was applied to 440 families of children with special needs. Participants consisted of 440 parents of children with a mean age of 7.9 ± 4.64 years (247 females, 193 males). The sample included 335 mothers and 105 fathers. The mean age was 36.12 ± 6.81 years for mothers and 39.74 ± 7.10 years for fathers. Demographic characteristics of the participants are given in Table 2.

**Table 2:** Demographics of the participants.

Characteristics (children) (n = 440) (mean age = 7.9 ± 4.64)		n	%
Sex	Female	247	56.1
	Male	193	43.9
Diagnoses	Cerebral palsy	278	63.2
	Muscular dystrophy	39	8.9
	Autism spectrum disorder, other common developmental disorders	53	12
	Genetic disorders	37	8.4
	Mental retardation	21	4.8
	Other (epilepsy, spina bifida, etc.)	12	2.7
Characteristics (parents)	Mother (X ± SD = 36.12 ± 6.81)	335	76.1
	Father (X ± SD = 39.74 ± 7.10)	105	23.9
Education level of mothers	Illiterate	14	3.3
	Primary	142	32.3
	Intermediate	63	14.3
	Secondary	109	24.8
	University or higher	112	25.3
Education level of fathers	Illiterate	5	1.2
	Primary	104	23.6
	Intermediate	46	10.5
	Secondary	129	29.3
	University or higher	156	35.4

The reliability coefficient (Cronbach’s alpha) of the scale was obtained as 0.929 by applying item analysis to the 88-item scale. This value is quite high and shows consistency between the items in the scale. As a result of item analysis, it was seen that the reliability coefficient of the scale increased when items 26, 31, 32, 34, 37, 53, and 58 were excluded from the scale separately. Therefore, it was decided to remove these items from the scale. Since the item-total correlation of item 20 was less than 0.25, this item was also excluded from the scale. Thus, with item analysis, 8 items were removed from the scale and the 88-item scale was reduced to 80 items. The reliability coefficient of the 80-item scale increased from 0.929 to 0.958.

The scale, having been reduced to 80 items by item analysis, was examined in terms of a structure suitable for factor analysis. For this purpose, the KMO coefficient, which is a measure of sample adequacy for factor analysis, was used, and the determinant value of the correlation matrix was examined by Bartlett’s test of sphericity, which showed whether the correlation matrix was ​​equal to the identity matrix. The KMO value was obtained as 0.862 and Bartlett’s sphericity test value was significant (c2 = 25213.86, P < 0.001). These values showed that the sample met the criteria for factor analysis. In the initial EFA (PCA with varimax rotation), items having factor loadings greater than 0.45 were taken into account. The items that had factor loadings distributed in more than one factor with the difference between these loads being less than or equal to 0.10 were excluded from the analysis. After testing the 80 items, a total of 20 items were excluded from the scale, 19 of which (1, 3, 6, 8, 17, 25, 36, 39, 41, 42, 47, 50, 51, 55, 62, 65, 67, 75, 82) did not load on any factor (<0.40) and one of which (item 56) was distributed in two factors with the difference being less than 0.10. In the second round, the factor loadings of 11 items (2, 7, 9, 13, 19, 30, 33, 35, 46, 64, 69) were less than 0.45 and one item (item 80) had a difference of less than 0.10. In the final factor analysis, all of the remaining 48 items had factor loadings greater than 0.45, ranging from 0.49 to 0.77. The EFA confirmed a four-factor structure explaining 49% of the total variance. The items that loaded on the four factors were examined in terms of content and named as awareness (F1, 18 items), attitude and behavior (F2, 16 items), social participation (F3, 8 items), and engagement in rehabilitation (F4, 6 items). Table 3 provides English translations of the original Turkish items and their factor loadings.

**Table 3:** Factor loadings of the items of the questionnaire.

	Items	F1	F2	F3	F4
77.	I am aware of my responsibilities in the rehabilitation of my child.	0.771			
70.	I notice changes in my child’s physical development.	0.697			
72.	I believe that rehabilitation should be regular and continuous.	0.696			
59.	I will do my best to ensure that my child receives a good rehabilitation service.	0.650			
73.	I wonder and learn about my child’s rehabilitation process.	0.649			
66.	I recognize the risks in the environment for my child and take the necessary precautions.	0.644			
71.	Healthcare professionals are aware of the importance I attach to rehabilitation and can count on me.	0.629			
79.	I don’t understand what my child means.	0.616			
61.	I provide the conditions for my child’s self-care needs.	0.610			
74.	I understand and apply the suggestions of the healthcare professionals regarding the rehabilitation of my child.	0.604			
48.	I express my positive or negative thoughts to healthcare professionals and I would like to receive feedback.	0.579			
52.	I use clear statements to inform healthcare professionals about the general situation of my child.	0.575			
57.	I believe in the necessity of rehabilitation programs.	0.569			
78.	In accordance with our economic situation, I select and use necessary tools, equipment, materials, and so on for rehabilitation program.	0.544			
81.	I think my child can do a lot of things.	0.543			
68.	I know the duties of healthcare professionals and the aims of rehabilitation practices.	0.521			
45.	I need information from my rehabilitation specialists about my responsibilities.	0.520			
76.	I do not think my child needs to be supported in terms of social participation.	0.503			
12.	I try to understand and support my child’s difficulties.		0.746		
11.	I can’t be consistent and determined with my child.		0.690		
10.	I try different games with my child and help him/her learn with fun.		0.689		
14.	I try to increase my child’s independence in line with his/her abilities.		0.681		
21.	I give my child the opportunity to do his/her daily activities or tasks that require skills on his/her own.		0.666		
16.	I believe I can understand child and put myself in my his/her shoes.		0.665		
23.	When communicating with my child, I keep in mind that he/she is an individual and a part of society.		0.661		
15.	I allow my child to develop him/herself by supporting his/her sense of accomplishment.		0.639		
43.	I support my child to participate in activities by communicating with family members.		0.598		
54.	Our communication is good when we are with my child.		0.591		
22.	I’m being impatient with my child.		0.570		
38.	I understand and accept my child’s disability.		0.545		
44.	I inform my child about his/her health and give him/her necessary explanation.		0.543		
18.	I pay attention to my behavior by being aware that I am a role model for my child.		0.535		
4.	I make environmental arrangements to support the physical capacity of my child.		0.522		
5.	I allow my child to spend time with his/her peers.		0.514		
84.	I am interested in activities that will improve my child’s sociality and increase his/her social participation.			0.773	
87.	I organize my child’s social life in accordance with the goals of the rehabilitation program.			0.727	
88.	I direct my child to activities such as sports and hobbies in accordance with his/her current situation.			0.720	
86.	I support my child’s participation in activities appropriate to his/ her health condition.			0.696	
49.	I ensure that my child participate in training activities in groups.			0.628	
83.	I attend organizations such as conferences, seminars, and scientific meetings related to my child’s health condition.			0.572	
85.	I have limited participation in social activities due to my child’s health condition.			0.568	
63.	I try to choose educational toys that support my child’s development.			0.555	
27.	I attend my child’s rehabilitation sessions on time and regularly.				0.707
40.	I spare necessary time for my child to participate in the rehabilitation program.				0.690
24.	I actively participate in my child’s rehabilitation sessions and follow them.				0.689
29.	I can’t follow the home program given by the rehabilitation specialist.				0.582
28.	Family members do not actively participate in my child’s rehabilitation program.				0.571
60.	I provide the necessary conditions for my child’s healthy diet.				0.492

Two hundred subjects of the sample group were retested to determine the reliability after 2 weeks and the Pearson correlation coefficient between the two applications was found to be 0.772 (P = 0.001). The internal consistency coefficient was also used in the reliability analyses of the questionnaire. The Cronbach alpha of the subdomains ranged from 0.799 to 0.912, and the Cronbach alpha of the questionnaire was found to be 0.943. Consequently, the FFQR showed valid and reliable scores of family functioning within the rehabilitation context.

## 4. Discussion

In recent years, when studies about the rehabilitation programs of children with special needs are examined, the accepted approaches are intervention methods in which the therapists and the families cooperate, seeing the child and the family as a whole and determining the targets in this direction. For these children who spend the majority of their time with their families, the contribution of the family is important in ensuring that rehabilitation is effective, permanent, and adaptable to daily life [15,24–27]. Family-centered research and practices, which emphasize the importance of the involvement of the family in the evaluation and intervention process in the rehabilitation of children with special needs, is gradually increasing. However, there is a lack of measuring the contribution of the family in this process. 

When the literature is examined, it is seen that family participation in rehabilitation is particularly evaluated in goal-setting and decision-making processes [28–30], and qualitative research techniques such as the interview method are mostly used in these evaluations. It was found that the methods used are not structured and do not have standardization [31–33]. There is a need for a standardized, reliable, and valid measurement tool to determine the level of functioning. This study reports a comprehensive, self-reported measurement tool developed to measure family functioning in the rehabilitation programs of children with special needs: the Family Functioning Questionnaire in Rehabilitation (FFQR). The FFQR, which consists of four main domains, showed adequate psychometric properties of family functioning within the rehabilitation context.

The needs and sociocultural characteristics of society play a role in structuring scales. Therefore, it is important to evaluate functioning with scales developed based on the experiences of individuals working in the relevant field. In the present study, after determining the need for measuring family functioning, the items of the questionnaire were created by interviewing rehabilitation professionals accordingly and these items were presented to experts in the field. Thus, it was assured that the questionnaire reflected family functioning better by considering the experiences of rehabilitation staff. 

The FFQR is a new measurement tool available for healthcare researchers and practitioners. 

The tool assesses the family function in four different dimensions and a separate score that can be calculated for each subdimension. It has a general content applicable to the families of children with special needs who have different diagnoses. With its large sample size, mixed-type design and good psychometric properties, this scale is an important value in family-centered research.

Although the current questionnaire fills an important gap in the field of rehabilitation with its comprehensiveness, there are still some limitations. First, the FFQR is a self-reported questionnaire and it may be difficult to predict the accuracy of the results due to the social desirability bias phenomenon. Second, during the development of the questionnaire, real-life conditions and the child and family’s natural environments were not observed and the views of families were not included. Further studies are needed in which the results are supported by qualitative interview methods and family views are also evaluated. It is also suggested that a therapist version of the questionnaire be developed so that therapists working with children with special needs can evaluate the functioning of the family in rehabilitation.

In conclusion, the FFQR was developed as a questionnaire with sufficient validity and reliability that can be used in the rehabilitation field. It can be used in studies involving family training interventions and the inclusion of the family as a part of the rehabilitation process. With the FFQR, comparative studies examining factors such as age group, diagnosis, and severity of the disease and studies measuring the functioning level of families from different socioeconomic levels can be designed.

## Acknowledgment/Disclaimers/Conflict of interest

The present study was funded by the Scientific and Technological Research Council of Turkey (TÜBİTAK) under the scope of the 2211/A Domestic PhD Scholarship Program.

This study was presented as a poster presentation at the 28th Annual Meeting of the European Academy of Childhood Disability (EACD), 1–4 July 2016, Stockholm, Sweden.
